# SNAPPE II Score as a Predictor of Neonatal Mortality in NICU at a Tertiary Care Hospital in Pakistan

**DOI:** 10.7759/cureus.20427

**Published:** 2021-12-15

**Authors:** Amin Ali, Shabina Ariff, Roshanara Rajani, Waqar H Khowaja, Abdul Lateef Leghari, Sher Wali, Rahil Barkat, Anum Rahim

**Affiliations:** 1 Neonatology and Pediatrics, Dow University of Health Sciences, Karachi, PAK; 2 Pediatrics and Child Health, Aga Khan University Hospital, Karachi, PAK; 3 Neonatology, Aga Khan University Hospital, Karachi, PAK; 4 Pediatrics, Aga Khan University Hospital, Karachi, PAK; 5 Research, Aga Khan University, Karachi, PAK; 6 Indus Hospital Research Center, The Indus Hospital, Karachi, PAK

**Keywords:** risk, scoring, snape ii, predictors, neonatal mortality

## Abstract

Introduction

The concept of illness severity scoring has been around for long and is currently being utilized in many neonatal intensive care unit (NICU). Scoring systems that help to quantify mortality risks on the basis of clinical conditions not only help in estimating prognosis, but also help clinicians in making decisions particularly in situations presenting with dilemmas. This study aims to determine SNAPPE-II (Score for Neonatal Acute Physiology-Perinatal Extension) score as a predictor of neonatal mortality in NICU at a tertiary care hospital in Pakistan.

Methodology

It was a longitudinal cohort study. The study was conducted at a neonatal intensive care unit (NICU) of Aga Khan University Hospital (AKUH) Karachi, Pakistan. All neonates were included who were born in AKUH and who needed respiratory support in NICU.

Results

A total of 333 newborns were enrolled for this study. Out of those 30 (9.1%) neonates expired while 298 (90.9%) survived. Area Under the Receiver operative curve was calculated to obtain the SNAPPE-II score’s diagnostic discrimination ability. Area under the curve (AUC) was 80.2±4.6% which corresponds to a moderate diagnostic accuracy for the prediction of neonatal mortality. The 95% CI for this was between 71.1-89.2%. SNAPPE-II category III (>40) was found to be the strongest predictor of mortality, with a sensitivity of 40% and a specificity of 98.7%.

Conclusion

The SNAPPE-II scoring system, we conclude, might be a valuable technique for predicting newborn death in resource-constrained NICUs.

## Introduction

The concept of illness severity scoring has been around for long and is currently being utilized in many neonatal intensive care unit (NICU) [1]. Scoring systems that help to quantify mortality risks on the basis of clinical conditions not only help in estimating prognosis, but also help clinicians in making decisions particularly in situations presenting with dilemmas [2]. Some of the scoring systems that are globally used include: CRIB (Clinical Risk Index of Babies), CRIB-II (Clinical Risk Index of Babies-II), SNAP (Score for Neonatal Acute Physiology), SNAP-II (Score for Neonatal Acute Physiology-II), SNAPPE (Score for Neonatal Acute Physiology-Perinatal Extension) and SNAPPE-II (Score for Neonatal Acute Physiology-Perinatal Extension-II) [1]. One of these scores is the SNAPPE-II scoring system, established by Richardson et al. which uses neonatal illness severity indices to forecast the rate of mortality and the length of the stay of newborns in NICU [3].

The assessment of morbidity and mortality using such scores also plays a significant role in estimating standard of care among different institutes. Although readily available, demographics like weight at birth, gestational age, and gender are not important indicators of morbidity. The predecessor of SNAPPE-II, i.e. SNAP (Score for Neonatal Acute Physiology), which was established in 1993, was for babies of all birth weights and validated as a predictor of mortality, morbidity, and resource utilization, and was a score based on physiological values despite using commonly accessible vital signs and laboratory test values, but it consisted of a total of 34 variables [4-8]. The Clinical Risk for Babies score, which was made for neonates less than 1.5 g, takes into account three physiologic variables additionally, i.e., weight at birth, gestational age, and congenital anomalies [9]. Studies have not only validated CRIB score as an anticipation of mortality [9] and morbidity [10], but these studies have also been replicated [11]. The modification of neonatal risk scores has been studied in the past [12], as well as the utilization of SNAP and CRIB in their initial years of usage [13]. The issue with the widespread use of these first-generation neonatal mortality scores was the limitation associated with them. SNAP was difficult to use due to the extensive number of variables and the complexity of items, while CRIB was inapplicable to infants born outside the hospital. Thus Richardson et al. developed the SNAPPE-II scoring system, a modified simpler version of SNAP score. Only nine criteria are recorded in this score: Average/mean blood pressure, PO2/FiO2, lowest temperature (ºF), serum-pH, numerous seizures, urinary output, newborn weight, Apgar score, and little for gestational age.

To the best of our knowledge, no such tool has been tested in the context of Pakistan to predict the neonatal mortality. Our study will form the basis of data on neonatal mortality scores and provide an insight towards the future utilization of SNAPPE-II scores in our institute. Thus, this study aims to determine SNAPPE-II score as a predictor of neonatal mortality in NICU at a tertiary care hospital in Pakistan. 

## Materials and methods

A prognostic prediction model is created to produce estimates of the risk of the presence/occurrence or likely outcome of a specific patient prognosis using multiple clinical or non-clinical characteristics in order to aid individualize diagnostic and treatment decision-making in healthcare. A longitudinal cohort study is the ideal strategy for prognostic studies. Participants will be included in the cohort at a certain time period and will be tracked over time in order to predict the desired outcome or occurrence. The study was conducted at the Neonatal Intensive Care Unit (NICU) of Aga Khan University Hospital (AKUH) Karachi, Pakistan. Ethical approval of this study was taken from the ethical representative committee (ERC) of AKUH.

Eligibility criteria

All neonates were included who were born in AKUH and who needed respiratory support in NICU. Neonates with life-threatening surgical conditions such as complex congenital heart and surgical conditions were excluded from the study.

Sample size and sampling technique

The sample size was estimated to test the association of SNAPPE-II score and neonatal mortality using chi-square test of association. Literature suggests that the proportion of dead neonates is different across three risk categories (i.e., mild, moderate and severe) of SNAPPE-II score. The null hypothesis assumed proportion of death is the same across three categories while the anticipated proportion of mortality is taken as an alternate hypothesis from the above-mentioned reference. The sample size of 333 neonates would require the power of 80% and the 95% confidence interval. The sample size was estimated using GPower software. To enroll participants, a non-probability consecutive sampling technique was used. Data were collected from neonates admitted to NICU of AKUH meeting the eligibility criteria during the designated study period.

Data collection

Before data collection, informed consent was taken from parents or caregivers. All data were collected within 48 hours of birth using a predesigned proforma. The data collected from the neonates included: mean blood pressure, PO2/FiO2, lowest temperature (ºF), serum-pH (lowest serum pH within 24 hours of admission), multiple seizures (>1 seizure within 12 hours of admission), urinary output, weight at the time of birth, Apgar score and gestational age. The correlation between SNAPPE-II scores in the first 48 hours after birth and the mortality rate in babies admitted to NICU was assessed. All data entered were kept in complete confidentiality.

Data analysis procedure

Descriptive statistics were calculated for independent variables. Mean and standard deviation were calculated for quantitative variables, and proportion was calculated for categorical variables. P-value of 0.10 was considered significant at univariate analysis or variables that were insignificant but have biological plausible relationship with dependent variable were kept for the final model. Multiple Logistic Regression was applied to predict the association between SNAPPE-II scores and mortality. The ability of the SNAPPE-II score to predict neonatal death was evaluated using a Receiver Operating Characteristics (ROC) curve. The best cut-off score for predicting death was determined by visual inspection of the curve at a level that combined maximum sensitivity and optimal specificity. For various cut-off scores, positive predictive values (PPV) and negative predictive values (NPV) were determined. A P-value of less than 0.05 was used to determine statistical significance.

## Results

A total of 333 newborns were enrolled for this study. Out of those 30 (9.1%) neonates expired while 298 (90.9%) survived. Table 1 shows the maternal baseline characteristics of all deliveries. Out of all the ones who survived 81.5% were preterm births while 96.7% were preterm among those who did not survive. However, the difference in the gestational age at delivery was significantly high among both the groups (p=0.035). Overall 27.8% mothers suffered pregnancy-induced hypertension (PIH) during pregnancy, the ratio of which did not differ among the dead and alive neonate groups (p=0.370). Similarly, about 33% pregnancies suffered gestational diabetes which was also comparable among both the groups (p=0.490). A significantly higher 78.6% mothers of expired neonates received antenatal steroids (p=0.015). As mentioned in Table 1, the frequency of each mode of delivery was alike among both the groups (p=0.717). Interestingly the ratio of abnormality on antenatal Doppler was found to be statistically insignificant among both the groups (p=0.089).

**Table 1:** Maternal baseline characteristics λ - Fisher’s Exact test ¥ - Chi-square Test EL LSCS: Elective lower segment cesarean section; EM LSCS: Emergency lower segment cesarean section; SVD: Spontaneous vaginal birth.

Variable	Alive n (%)	Expired n (%)	Total n (%)	P-value
Gestational Age at delivery				
Term	55(18.5)	1(3.3)	56(17.1)	0.035^¥^
Preterm (<37 weeks)	242(81.5)	29(96.7)	271(82.9)	
Pregnancy-Induced Hypertension (PIH)				
Yes	79(28.5)	6(20.7)	85(27.8)	0.370^¥^
No	198(71.5)	23(79.3)	221(72.2)	
Gestational Diabetes Mellitus (GDM)				
Yes	93(33.6)	7(26.9)	100(33.3)	0.490^¥^
No	184(66.4)	19(73.1)	203(67.0)	
Antenatal Steroids given				
Yes	61(44.5)	11(78.6)	72(47.7)	0.015^¥^
No	76(55.5)	3(21.4)	79(52.3)	
Mode of delivery				
EL LSCS	240(85.4)	23(82.1)	263(85.1)	0.717^λ^
EM LSCS	8(2.8)	1(3.6)	9(2.9)	
SVD	33(11.7)	4(14.3)	37(12.0)	
Antenatal Doppler				
Normal	271(94.4)	24(85.7)	295(93.7)	0.089^λ^
Abnormal	16(5.6)	4(14.3)	20(6.3)	
Abnormal Doppler				
Reduced Flow	8(66.7)	0	8(57.1)	0.165^λ^
Absent Flow	2(16.7)	1(50.0)	3(21.4)	
Reversal End Diastolic flow	2(16.7)	1(50.0)	3(21.4)	

Among all the neonates 59.3% were male, the frequency of which was consistent among both the groups (p=0.477). Median APGAR scores at 1 and 5 minutes were each considerably lower among the expired neonates with <0.0001 p-value. Likewise, the median birth weight was found to be significantly higher, i.e., 1.7 kg among the survived neonates (p=<0.0001). About 66% expired neonates were given surfactant while only 23.7% survived neonates received the same (p=<0.0001). Considering the type of assisted ventilation 90% expired neonates were on mechanical ventilation while only 20.1% received mechanical support. High flow and oxygen support were required by about 29% each in the survived group, whereas none was on oxygen support in the expired group and only 3.3% received high flow. The difference was statistically significant for mechanical ventilation, high flow and oxygen support (p=<0.0001), while the frequency of continuous positive airway pressure (CPAP) did not differ among the groups. Surprisingly the days of assisted ventilation were insignificant among both the groups as shown in Table 2. Similarly, with p-values of 0.218 and 0.301, the frequency of apnea and total days in neonatal ICU were not statistically different in both groups.

**Table 2:** Neonatal baseline characteristics Ʈ- Independent T-Test ₼ - Mann-Whitney U test λ - Fisher’s Exact test ¥ - Chi-square Test *Median (Interquartile Range) ^Mean (Standard Deviation) CPAP: Continuous positive airway pressure; NICU: Neonatal intensive care unit.

Variable	Alive n (%)	Expired n (%)	Total n (%)	P-value
Gender of the baby				
Male	175(58.7)	19(65.5)	194(59.3)	0.477^¥^
Female	123(41.3)	10(34.5)	133(40.7)	
APGAR score at 1 min*	8.0(6.5-11.4)	4(3.0-7.2)	8.0(7.0-8.0)	<0.0001^₼^
APGAR score at 5 min*	9.0(7.5-13.6)	7.5(4.25-9.0)	9(7.5-12.5)	<0.0001^₼^
Birth weight (kg)*	1.7(1.4-2.4)	1.0(0.6-1.7)	1.7(1.3-2.4)	<0.0001^₼^
Use of surfactant				
Yes	65(23.4)	20(66.7)	85(27.6)	<0.0001^¥^
No	213(76.6)	10(33.3)	223(72.4)	
Assisted ventilation				
CPAP	63(21.1)	2(6.7)	65(19.8)	<0.0001^¥^
Mechanical Ventilation	60(20.1)	27(90.0)	87(26.5)	
High flow	88(29.5)	1(3.3)	89(27.1)	
Oxygen	87(29.2)	0	87(26.5)	
Days of ventilation				
CPAP^	3.1 ± 2.4	6.0 ± 5.7	3.1 ± 2.5	0.100^Ʈ^
Mechanical Ventilation*	3.0(2.0-4.0)	4.0(1.8-9.5)	3.0(2.0-5.0)	0.378^₼^
High flow*	2.0(1.0-2.0)	0	2.0(1.0-2.0)	0.092^₼^
Apnea				
Yes	8(2.8)	2(7.1)	10(3.2)	0.218^λ^
No	281(97.2)	26(92.9)	307(96.8)	
Total days in NICU*	7.0(4.0-10.0)	6.0(2.0-12.3)	6.0(4.0-10.0)	0.301^₼^

ROC curve analysis

Diagnostic analysis was performed in order to analyze the predictability of SNAPPE-II score for neonatal mortality. According to the literature the cutoffs were taken for mild with 0-20 score, moderate with 21-40 score and severe with >40 score. The distribution is expressed in Table 3. However, each category of SNAPPE-II score was significantly different among the two groups (p=<0.0001).

**Table 3:** SNAPPE-II score categories λ - Fisher’s Exact test b - Subset of categories whose column proportion differs significantly.

Categories	Alive	Dead	Total	P-value
Category I -Mild (0-20)	271(90.9)	10(33.3)^b^	281(85.7)	<0.0001^λ^
Category II -Moderate (21-40)	23(7.7)	8(26.7)^b^	31(9.5)	
Category III -Severe (>40)	4(1.3)	12(40.0)^b^	16(4.9)	

**Figure 1:**
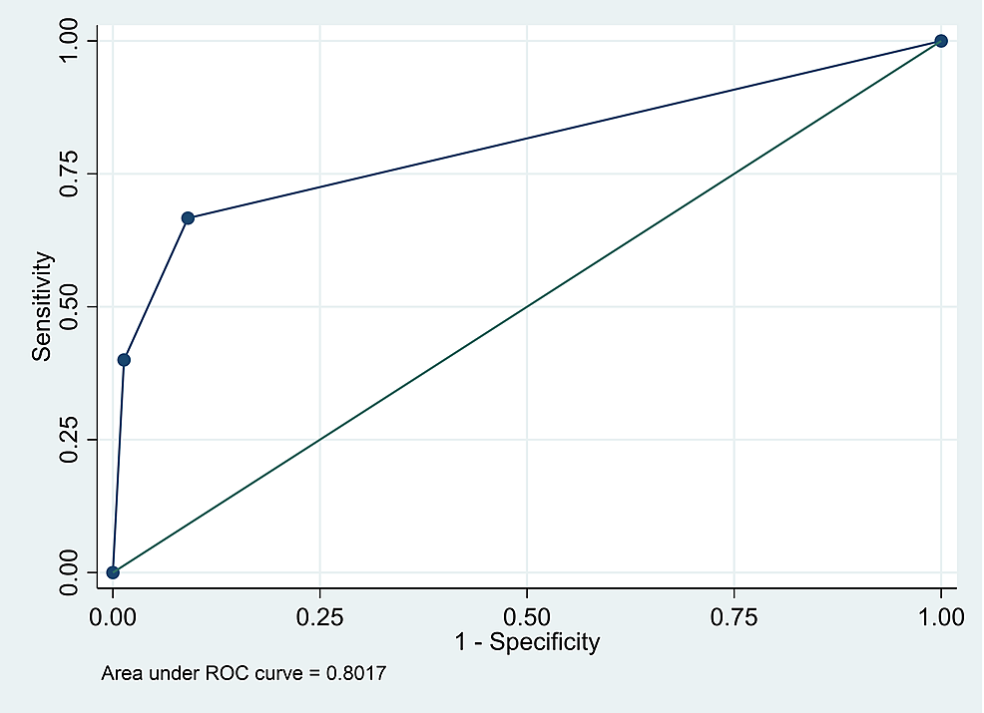
ROC curve (AUC) for SNAPPE-II score categories ROC: Receiver operating characteristic; AUC: Area under the curve.

Area Under the Receiver operative curve was calculated to obtain the SNAPPE-II score’s diagnostic discrimination ability, the results of which were as follows. Area under the curve (AUC) was 80.2±4.6% which corresponds to a moderate diagnostic accuracy for prediction of neonatal mortality. The 95% CI for this was between 71.1% and 89.2% (Figure 1).

The diagnostic analysis was performed for comparison amongst the categories. Firstly, category I was compared with category II and III, the sensitivity of which was 66.7% while the specificity of that was 90.9%. Interestingly when category I and II was compared with category III, the specificity increased up to 98.7% while the sensitivity decreased as low as 40.0%. The diagnostic accuracy of both the comparisons was 88.7% and 93.3%, respectively. Since the sensitivity itself decreased from the above comparisons, the effect strength of sensitivity also decreased as shown in Table 4.

**Table 4:** Diagnostic analysis - SNAPPE-II score and mortality status

	(I vs II & III)	(I & II vs III)
Sensitivity (%)	66.7	40.0
Specificity (%)	90.9	98.7
Positive Predictive Value (%)	42.6	75.0
Negative Predictive Value (%)	96.4	94.2
Diagnostic Accuracy (%)	88.7	93.3
Effect strength of sensitivity (%)	57.61	38.7

## Discussion

The SNAPPE-II scoring system merges biochemical and physiologic function testing. In this study, 333 newborns in the NICU were prospectively enrolled to see if there was an association between SNAPPE-II score and mortality. Our study found that the infant mortality rate was 9.1%, which is significantly lower than 26.4% [14] and 38% [15] found in studies by Ashrafzadeh et al. and Vasudevan et al., respectively. In agreement to the result of our study, the neonatal mortality rate ranges from 4.3% to 11% in Canada [16]. According to our study, the percentage of neonatal mortality is higher in SNAPPE-II category III (>40) followed by SNAPPE-II category II (21-40). This finding is also supported by the study and reported that the greater the score of SNAPPE-II, the greater was the mortality risk of newborns [17]. According to a study conducted by Jain and Bansal [18] and Ramirez et al. [19], SNAPPE-II scores of 40 and higher were related with greater neonatal mortality.

The ROC in this study was 0.82, indicating a strong association between the severity of the score and the neonatal fatality rate. According to a cohort study carried out by Aryana et al., the score of SNAPPE-II is a good predictor of mortality in NICU newborns, with an ROC of 0.92 (a strong relationship between the score and mortality rate) [20]. However, another study conducted by Rachuri et al. found an ROC of 0.622 indicating a moderate relationship between the score severity and mortality rate [21].

In this study, SNAPPE-II category III (>40) was found to be the strongest predictor of mortality, with a sensitivity of 40% and a specificity of 98.7%. One research study found that SNAPPE-II had a sensitivity of 78.8% and a specificity of 47% [21], while another study reported a sensitivity and specificity of 94% and 83%, subsequently [22]. According to our findings, the PPV and NPV of the SNAPPE-II score were 75 and 94.2%, respectively. In other words, approximately 75% of newborns with high scores perished, while 94% of babies with low scores lived. Another study also reported that SNAPPE-II had a PPV of 58.9% and NPV of 93.41%.

Our study's strength is that we excluded all babies with life-threatening surgical diseases such as complex congenital heart disease and surgical conditions that, regardless of the SNAPPE-II score, have risk factors for mortality. The limitation of our study is that the subsequent clinical course was not taken into account because the data were obtained within 48 hours of life. As a result, it fails to accurately forecast the prognosis in preterm newborns who will develop nosocomial infections and neonates whose internal condition is changing dynamically.

## Conclusions

SNAPPE-II should be utilized as an institutionalized management practice to assess the seriousness of sickness and prognosis. It could also help prioritize the treatment of unwell babies and provide disease severity counselling to their parents. The SNAPPE-II scoring system, we conclude, might be a valuable technique for predicting newborn death in resource-constrained NICUs.
